# Neurobehavioral manifestations of developmental impairment of the brain

**DOI:** 10.2478/v10102-010-0012-4

**Published:** 2010-06

**Authors:** Michal Dubovický

**Affiliations:** Institute of Experimental Pharmacology & Toxicology, Slovak Academy of Sciences, SK-84104, Bratislava, Slovakia

**Keywords:** neurobehavioral dysfunctions, brain development, cognitive disorder, mental disorder, emotional disorder, behavioral disorder, developmental neurotoxicity, environmental factors

## Abstract

Individual characteristics of human nature (*e.g.* introversion, extroversion, mood, activity, adaptability, aggressiveness, social ability, anxiety) do not need to be primarily innate. They can be determined by the action of various influences and their interactions on functional development of the brain. There is ample epidemiological and experimental evidence that chemical and/or physical factors acting during sensitive time windows of the brain development can cause mental, behavioral, emotional and/or cognitive disorders. Environmental pollutants, addictive substances, drugs, malnutrition, excessive stress and/or hypoxia-ischemia were reported to induce functional maldevelopment of the brain with consequent neurobehavioral disorders. The article provides review on most significant neurobehavioral manifestations of developmental impairment of the brain during prenatal, perinatal and early postnatal period. The most known adverse factors causing developmental neurobehavioral dysfunctions in humans as well as in experimental animals are discussed.

## Introduction

Introversion, extroversion, mood, activity, adaptability, distractibility, persistence and attention span, as well as aggressiveness, social ability, tendency to depression and/or anxiety do not need to be primarily innate. Individual characteristics of human nature and behavior could be determined by the action of various influences and their interactions on functional brain development.

There is ample epidemiological and experimental evidence that chemical and/or physical factors acting during sensitive time windows of the brain development can cause behavioral, emotional and/or cognitive dysfunctions. Environmental pollutants, addictive substances, drugs, malnutrition, excessive stress and/or hypoxia-ischemia were reported to induce functional maldevelopment of the brain with consequent neurobehavioral disorders.

The range of neurobehavioral alterations, however, depends on the intensity/dosage and duration of the factor in question as well as on the developmental stage of the brain. Chronic alcohol consumption during pregnancy results in fetal alcohol related abnormalities (FARA). Most serious manifestation of FARA is fetal alcohol syndrome (FAS). FAS is characterized by triads of pathological signs: prenatal and postnatal growth retardation, impairment of the CNS and facial deformities. On the other hand, mild social drinking in pregnancy causes mostly functional alterations of the brain, called alcohol related neurodevelopmental disorders (ARND) or fetal alcohol effect (FAE). FAE is characterized by verbal, cognitive and attention deficit (McGough *et al*., 2009; McGee *et al*., 2009).

Fetal and neonatal brain development is characterized by developmental time windows during which certain brain regions or neuron types are specifically sensitive to environmental influences. The immature brain is much more sensitive to abnormal experience, particularly sleep deprivation, drug exposure, and maternal separation. The critical time period, during which features in brain susceptibility to such experience change, however, has not yet been fully determined. Basal forebrain cholinergic neurons were found to be sensitive to nerve growth factor (NGF) deprivation during the first postnatal week not later (Molnar *et al*., 1997). The study of Feng *et al*. (2001) is an excellent example of sensitive developmental periods from the neurotoxicity point of view. Male rats neonatally treated with the tricyclic antidepressant clomipramine (CLI) during various treatment windows, i.e. postnatal days (PD) 12–17, 14–20, 16–22, and 12–15, were in adulthood tested for sexual behavior. The rats treated with CLI showed significant sexual impairment in treatment windows PD12–17 and PD14–20 and slight sexual deficiency in the short window PD12–15. No significant sexual impairment was found in window PD16–22. The results indicate that PD14–20 is the latest window during which CLI treatment can produce adult sexual deficiency and that 6 days might be the shortest treatment window to produce significant behavior abnormalities.

Moreover, genetic disposition of the fetus, gender and racial differences may affect the action of individual factors and/or their interferences. Experimental neonatal stress and/or monosodium glutamate (MSG) treatment can be an example referring to gender issue. MSG and combined stressful stimuli represented by 10% NaCl administration and mild handling on PD 2, 4, 6, 8 and 10 were found to increase locomotor activity and slow down habituation in an open field test in adult male but not female rats (Dubovický *et al*., 1997; 1999).

Activity of enzyme alcohol-dehydrogenase metabolizing ethanol is different in Whites compared to Chinese, Native Americans and Japanese (Agarwal and Goedde, 1986). In this respect it is possible that fetal susceptibility to alcohol may also be dependent upon maternal racial and genetic dispositions interacting with consumption patterns. The basis of these differences remains however to be elucidated. Among Blacks, slower metabolism of nicotine was found in comparison with Whites (Benowitz *et al*., 1999). Perera *et al*. (2004) found higher umbilical cord serum of the nicotine metabolite cotinine in Blacks versus Hispanic newborns. Racial differences in nicotine metabolism could reflect differences in low-level prenatal smoking on infant behavior. While among White infants, increased cotinine levels were associated with increased arousal and excitability and decreased self-regulation (ability to calm dawn), on the contrary, in Black infants increased cotinine levels were associated with decreased arousal and excitability and increased ability to self-regulate (Yolton *et al*., 2009).

The present article provides review on the most significant neurobehavioral manifestations of developmental impairment of the brain during the prenatal, perinatal and early postnatal/neonatal period. The best known adverse factors causing neurodevelopmental behavioral dysfunctions in humans as well as in experimental animals with possible mechanisms of action are discussed. The classification of neurobehavioral disorders is not unequivocal. Individual manifestations of the disorders can overlap. Attention deficit-hyperactivity disorder (ADHD) generally belongs to the group of behavioral disorders. However, attention deficit as its part can be classified as a cognitive dysfunction and ADHD may be accompanied also by mood changes. To facilitate orientation in the broad spectrum of neurobehavioral disorders, the review is divided into four most important groups of disorders: serious neurodevelopmental disorders, cognitive disorders, mental disorders and emotional and behavioral disorders.

## Serious neurodevelopmental disorders

The majority of signs of serious neurodevelopmental disorders do not belong to the spectrum of neurobehavioral dysfunctions. Although some of these disorders can be associated with neurobehavioral deficit or with an increased risk for behavioral alterations in later postnatal development. However, if cerebral palsy, neurological disorders, epilepsy and severe mental retardation were not mentioned, the survey of significant neurodevelopmental pathologies would be incomplete.

**Cerebral palsy** describes a group of permanent disorders of the development of movement and posture, causing activity limitations attributed to nonprogressive disturbances occurring in the developing fetal or infant brain. The motor disorders of cerebral palsy are often accompanied by disturbances of sensation, perception, cognition, communication, and behavior, or by epilepsy and secondary musculoskeletal problems (Horsman *et al*., 2010). While in certain cases there is no identifiable cause, other etiologies include problems in intrauterine development (*e.g.* exposure to **radiation, infection**), **hypoxia** of the brain and **birth trauma** during labor and delivery, and **complications in the perinatal period** or **during childhood** (Brucknerová *et al*., 2008). After birth, other causes include **toxins**, severe **jaundice**, **lead poisoning**, **physical brain injury**, incidents involving **hypoxia** to the brain and **encephalitis** or **meningitis** (Mendola *et al*. 2002; Gilbertson, 2004).

A **neurological disorder** is a disorder of the body's nervous system. Structural, biochemical or electrical abnormalities in the brain or spinal cord or in the nerves leading to or from them, can result in manifestations such as paralysis, muscle weakness, poor coordination, loss of sensation, seizures, confusion, pain and altered levels of consciousness. A unique example of neurological complications due to developmental effects of chemicals is the Minamata disease. The first well-documented outbreak of **acute developmental methyl mercury poisoning** by consumption of contaminated fish occurred in Minamata, Japan, in 1953. Typical features of the disease are as follows: sensory disturbances, ataxia, seizures, constriction of the visual field, auditory disturbances and tremor (Harada, 1995, Ekino *et al*., 2007). Fetal neurodevelopment depends on cell programs, developmental trajectories, synaptic plasticity, and oligodendrocyte maturation, which are variously modifiable by factors such as stress and **endocrine disruption**, exposure to **pesticides** such as **chlorpyrifos** and to **drugs** such as **terbutaline**, and by **premature birth** (Mendola *et al*., 2002; Connors *et al*., 2008). Prenatal **nicotine** was reported to cause abnormal reflexes and hypertony/hypotony in neonates and infants (Yolton *et al*., 2009).

**Epilepsy** is a common chronic neurological disorder characterized by recurrent unprovoked seizures. These seizures are transient signs and/or symptoms of abnormal, excessive or synchronous neuronal activity in the brain. Epilepsy is more likely to occur in young children or people over the age of 65 years, however it can occur at any time (Fisher *et al*., 2005). During the neonatal period and early infancy the most common causes of epilepsy include **hypoxic-ischemic encephalopathies, CNS infections, toxins, trauma, congenital CNS abnormalities** and **metabolic disorders** (Scher, 2003; Vestergaard *et al*., 2005).

**Mental retardation** is a generalized disorder, characterized by significantly impaired cognitive functioning and deficits in adaptive behavior with onset before the age of 18. It has historically been defined by an intelligence quotient score under 70. Once focused almost entirely on cognition, the definition now includes both a component relating to mental functioning and one relating to the individual's functional skills in the environment (Van der Aa *et al*., 2010). **Down syndrome, fetal alcohol syndrome** and **fragile X syndrome** are the three most common inborn causes of mental retardation. A woman who drinks **alcohol** or gets an **infection** like **rubella** during pregnancy may also have a baby with mental disability (Batshaw, 1993; Devenny *et al*., 2000; Merrick *et al*., 2006). If a baby has problems during **labor** and **birth**, as *e.g.* not getting enough **oxygen**, he or she may have developmental disability due to brain damage (Kaindl *et al*., 2009).

## Cognitive disorders

**Cognitive disorders** are disorders in which the central feature is impairment of memory, attention, perception, thinking, problem solving and language.

Drinking of **alcohol** during pregnancy seriously affects neurobehavioral development of children. Even social drinking in pregnancy was found to result in cognitive dysfunctions accompanied by deficit in language ability in infants both in reception and expression of the language (McGee *et al*., 2009). Attention problems were found in children of mothers drinking in pregnancy and/or using **psychotropic drugs** (Dalen *et al*., 2009).

**Polybrominated diphenyl eters (PBDEs)** are synthetic flame retardants added to polymers for the manufacture of electrical appliances, carpets and polyurethane foam. They have similar effects as polychlorinated biphenyls. They bind to androgen and estrogen receptors and can injure **the activity of thyroidal hormones,** which play a significant role in neurodevelopmental processes such as timing of neural and glial proliferation, migration and differentiation, myelination, synaptic connectivity, development of dopaminergic and cholinergic system (Figueredo *et al*., 1993; Sawin *et al*., 1998; Auso *et al*., 2004;). The functional maldevelopment due to PBDs can lead to neurocognitive dysfunctions. A mixture of PBDs in the neonatal period was reported to result in cognitive deficit with disorders of attention and inhibitory control in rats (Driscoll *et al*., 2009). Rats exposed prenatally to **polychlorinated biphenyls** and/or **methyl mercury** had a deficit in cognitive and behavioral tests (Sable *et al*., 2009).

About 12% of women do not quit smoking during pregnancy. **Nicotine** causes intrauterine growth retardation, increases the risk for miscarriage and cognitive and behavioral disorders. Nicotine mimics the action of acetylcholine which plays an important role in the development of auditory neural circuitry. During development, excessive stimulation of nicotine receptors can lead to their injury. This in turn can result in injury of sensory encoding of auditory information associated with disorder of auditory habituation in neonates, delayed vocalization, disorders of language perception and reading abilities in infants (Kable *et al*., 2009).

The Environment Protection Agency (USA) considers the blood level of 10 µg/dL **lead** to be a risk for health. Recent studies, however, showed that levels under 5 µg/dL can injure cognitive functions, lower the score of reading abilities and mathematics. Moreover, prenatal alcohol and/or drugs can increase sensitivity to lead toxicity in neonates and infants (Min *et al*., 2009).

Prenatal **cocaine** acts adversely during development by two basic mechanisms: it decreases uterine blood flow resulting in fetal hypoxia, and it affects development of the brain monoamines leading to functional maldevelopment of the brain mostly in the prefrontal cortex, which is responsible for executive functions, attention and neurocognitive functions. Prenatal cocaine causes attention deficit and has negative effects on reaction of the organism to new stimuli (Hurt *et al*., 2009; Eiden *et al*., 2009).

It is important to note that functional development of the brain does not end in infancy. Functional maturation of the brain continues to adolescence, *e.g.* processes of sprouting and pruning. **Alcohol** and **addictive substances** in adolescents can therefore have more deleterious effects than in adults. Binge drinking in adolescents was reported to cause macrostructural and microstructural changes of the white matter (*e.g.* volume, density of fibers, *etc.*) (Jacobus *et al*., 2009). These alterations in turn can lead to neurocognitive dysfunctions in later development or could potentate unfavorable effects of other environmental factors.

**Organophosphate pesticides** damage replication of neurons, differentiation, axogenesis, synaptogenesis and development of neuronal circuitry. They affect ACh and 5-HT systems, what in turn may lead to cognitive dysfunction as well as the emotional and behavioral disorders (Slotkin *et al*., 2009).

Prenatal and/or perinatal **hypoxia-ischemia** is a major factor for the development of cognitive dysfunctions in later life (Gitto *et al*., 2009; Brucknerová *et al*., 2008; Mach *et al*., 2009). Memory and learning deficits are very frequent consequences of lack of oxygen and nutrition during brain development. The hippocampus is one brain region that can be damaged and this site of damage has been implicated in two different long-term outcomes, cognitive memory impairment and the psychiatric disorder schizophrenia (de Haan *et al*., 2006). The risk of cognitive deficits is related to the severity of neonatal encephalopathy and the pattern of brain injury on neuroimaging, particularly the watershed pattern of injury (Gonzales & Miller, 2006). Cognitive deficit is one of the manifestations of fetal hydantoin syndrome due to teratogenicity of **phenytoin** (PHT), an anticonvulsant drug used in treatment of epilepsy. PHT is considered to induce teratogenicity by affecting the hemodynamic status of the pregnant mother as well as of the embryo/fetus, eventually leading to embryo-fetal hypoxia (Adams *et al*., 1990; Wells and Winn, 1996; Navarová *et al*., 2005; Ujházy *et al*., 2008). PHT causes serious structural and functional changes including memory deficiency (Okruhlicová *et al*., 2003; Ujházy *et al*., 2004; Mach *et al*., 2005). **Xenoestrogens** were reported to negatively affect spatial learning of male rats in water maze (Ceccarelli *et al*., 2009).

## Mental disorders

A mental disorder is a psychological or behavioral pattern associated with distress or disability that occurs in an individual and is not a part of normal development or culture. The recognition and understanding of mental health conditions has changed over time and across cultures, and there are still variations in the definition, assessment, and classification of mental disorders, although standard guideline criteria have been widely accepted (American Psychiatric Association, 2000). Mental disorders can arise from a combination of sources. In many cases there is no single accepted or consistent cause currently established. A common belief even to this day is that disorders result from genetic vulnerabilities exposed to environmental stressors.

**Schizophrenia** is a mental disorder characterized by abnormalities in the perception or expression of reality. It is most commonly manifested as auditory hallucinations, paranoid or bizarre delusions, or disorganized speech and thinking with significant social or occupational dysfunction. Onset of symptoms typically occurs in young adulthood, with around 0.4–0.6% of the population affected (Castle *et al*., 1991).

There is much evidence of neurodevelopmental origin of schizophrenia. Epidemiological studies found a **seasonally-related** increase in schizophrenia for people born in winter months when infections are more frequent (Battle *et al*., 1999). Reelin, a protein that regulates processes of neuronal migration and positioning in the developing brain, showed significantly reduced gene expression in the brain of schizophrenic patients as well as in animals used as amodel of this disease (Erbel-Sieler *et al*., 2004). In schizophrenia, dopaminergic hyperfunction in certain brain regions is considered the key element of the disease. The developmental model of schizophrenia has been proposed based on the dopamine hypothesis. It has been shown that neonatal exposure to the **dopamine D2/3 receptor agonist quinpirole** leads to supersensitivity or priming of D2/3 receptors (Kostrzewa, 1995; Kostrzewa *et al*., 2005). This supersensitivity was accompanied by spatial learning deficit and alterations in motor activity (Vorhees *et al*., 2009).

**Anxiety disorders** are blanket terms covering several different forms of abnormal and pathological fear and anxiety. Current psychiatric diagnostic criteria recognize a wide variety of anxiety disorders: generalized anxiety disorder, panic disorder, phobias, social anxiety disorder, obsessive–compulsive disorder, post-traumatic stress disorder, separation anxiety, childhood anxiety disorder. The amygdala is central to the processing of fear and anxiety, and its function may be disrupted in anxiety disorders.

There are many experimental studies confirming the relationship between prenatal/perinatal insults and adult anxiety disorders. Whereas in humans, more studies would be needed to ascertain long-term behavioral effects of adverse stimuli on pregnant women and their offspring. Emerging data from human and animal perinatal exposure studies demonstrate a subtle effect of **cannabis** upon later brain functioning including specific cognitive deficits, especially in visuospatial function, impulsivity, inattention and hyperactivity, depressive/anxiety signs and symptoms (Sundram, 2006). Maternal exposure to **caffeine** was found to induce long-term consequences on sleep, locomotion, learning abilities, emotivity, and anxiety in rat offspring (Nehlig & Debry, 1994).

**Hypoxia-ischemia** represents a risk factor for functional alterations of the brain structures and functions related to anxiety/fear. Asphyxiated animals in the perinatal period were found to exhibit a significantly decreased social aggressiveness and an increased social contact behavior, as well as increased anxiety levels in adulthood (Weitzdoerfer *et al*., 2004). Antenatal intermittent hypoxia caused a decrease of motor activity as well as an enhanced anxiety level in rats of both sexes, while males appeared to be more sensitive to hypoxic influence (Trofinmova *et al*., 2010). Other studies have also shown that neurobehavioral changes due to mild perinatal hypoxia were mainly related to emotional/anxious responses (Hoeger *et al*., 2000; Venerosi *et al*., 2006).

The neuroendocrine system, especially the hypothalamus-pituitary-adrenal (HPA) axis, is very sensitive to **excessive stressful stimuli** during development. Prenatal stress can interfere with functional development of the brain acan cause neurobehavioral dysfunctions in later postnatal period. Epidemiological studies provided knowledge on long-term effects of prenatal stress on neurobehavioral development (Graignic-Phillipe & Tordjman, 2009), cognitive ability and fearfulness in infancy (Bergman *et al*., 2007). Experimental studies showed that increased levels of stress hormones could affect development of hippocampus and other brain regions and cause long-term changes in HPA axis reactivity (Sapolsky *et al*., 1984; Uno *et al*., 1990). Behavioral alterations were correlated with an increase of corticostreone level in reaction to a stressful stimulus, as well as its prolonged secretion, and adecreased density of hippocampus glucocorticoid receptors (Henry *et al*., 1994; Maccari *et al*., 1995; Vallée *et al*., 1996; Maccari & Morley-Fletcher, 2007). Prenatal stress is associated with an increased anxiety/fear-like behavior in adult rats and an increased sensitivity of to stressful stimuli (Valleé *et al*., 1997). Repeated stress in late pregnancy in monkeys was found to induce depressive behavior in infant monkeys and cognitive deficit (Schneider, 1992). Prenatal social stress can program anxiety behavior and HPA axis responses to stress in offspring. Attenuated glucocorticoid feedback mechanisms in the limbic system may underlie HPA axis hyper-reactivity to stress in offspring (Brunton & Russel, 2010). Prenatal stress has been found to affect brain development and behavior of male and female rats differentially (Weinstock, 2007). On the other hand, early postnatal stress in the form of manipulation (handling) resulted in decreased anxiety and corticosterone secretion in reaction to stressful stimuli (Valée *et al*., 1997). Altered behavior observed in adulthood is likely the result of neurodevelopmental perturbations elicited by early life stress. It can be assumed that there is "a critical period" for neural circuits involved in emotional expression that contribute to lifelong susceptibility to stress (Matsumoto *et al*., 2009). Moreover, developmental administration of some drugs can affect HPA axis functioning in adulthood. Exposure to phenytoin in utero was found to increase catecholamine and corticosterone concentrations in response to a mild stressor in adult offspring (Makatsori *et al*., 2005).

**Mood/affective disorders** are characterized by a consistent, pervasive alteration in mood, and affecting thoughts, emotions, and behaviors. There are two basic types of mood disorders, i.e. depressive and bipolar disorder. In affective disorders, we also need to rely on experimental studies.

It was shown that prenatal exposure to the inhibitor of serotonin (5-HT) synthesis **para-chlorophenylalanine** (PCPA) caused fetal 5-HT depletion and change both in open field activity and in depression-related behavior in the adult rat offspring (Vataeva *et al*., 2008). Prescription of **selective serotonin reuptake inhibitors** and/or **serotonin-noradrenalin reuptake inhibitors** (SSRI/SNRI) in pregnancy and lactation may represent arisk for the unborn child. Prenatal administration of **fluoxetine** in mice resulted in dose-dependent development of affective disorders (Noorlander *et al*., 2008). Prenatal exposure to fluoxetine caused adecrease in a number of neurons in the frontal cortex (Swerts *et al*., 2009) and decrease in 5-HT receptor density in the frontal cortex in pre-pubescent rats and in the middle brain in adult rats (Cabrera-Vera *et al*., 1997). Generally, administration of SSRI and/or SNRI drugs during sensitive developmental periods of the brain increases the risk for neuronal circuitry alterations and maladaptive behavior persisting up to adulthood in the form of enhanced depression and/or anxiety, and even aggressive behavior (Borue *et al*., 2007).

## Emotional and behavioral disorders

Emotional and behavioral disorders form a broad category, which is used commonly in educational settings, to group a range of more specific perceived difficulties of children and adolescents. Both general definitions as well as actual diagnosis of these disorders may be controversial as the observed behavior may depend on many factors.

Emotions and behavior are controlled by a complex of central executive and regulatory functions. Executive functions are a complex of cognitive and behavioral competences inevitable in solving demanding, non-routine and aim-oriented tasks and/or situations. (organizational and information processing, oriented attention, working memory, inhibition). The emotional and behavioral regulatory system has two basic components. The first is the latency and intensity of reactions to environmental stimuli (reactivity), the second is represented by behavioral responses and strategy/coping modulated by the reactions (regulatory components) involving self-calming, communicative behavior, behavioral strategy to disperse attention from the incentive stimulus and/or approach/withdrawal behavior. Emotional arousal regulation provides excitatory/inhibitory balance, which protects central executive functions against excessive stimulation and helps to coordinate cortical systems. The dorsal and lateral prefrontal lobe, amygdala and other brain regions are responsible for these functions (Drevets and Raichle, 1998; Avants *et al*., 2007). **Prenatal cocaine** inhibits re-uptake of DA, NA and 5-HT on the presynaptic membrane and causes vasoconstriction followed by hypoxia. This can lead to long-term cognitive and neurodevelopmental consequences manifested in middle infancy, puberty or adolescence when there are increased demands, challenges and more complex cognitive processes are needed for educational, behavioral, social and executive functions. It has been shown that prenatal cocaine affects executive functions manifested by lowered inhibitory control of excessive verbal responses in children (Rose-Jacobs *et al*., 2009). Moreover, prenatal cocaine damages the prefrontal cortex and amygdale, which leads to cognitive and emotional dysfunctions manifested by memory and attention alterations (Li *et al*., 2009). Prenatal cocaine can affect these regulatory systems and results in attention disorder, frustration, impulsivity, disruptive behavior, low motor inhibition. In experimental animals prenatal cocaine resulted in stress-related freezing and aggressive behavior (Eiden *et al*., 2009).

**Autism spectrum disorders** (ASD) are a spectrum of psychological conditions characterized by widespread abnormalities of social interactions and communication, as well as severely restricted interests and highly repetitive behavior. Autism is a disorder of neural development characterized by impaired social interaction and communication, and by restricted and repetitive behavior. These signs all begin before a child is three years old. Autism affects information processing in the brain by altering nerve cell and synaptic connection and organization. The other two disorders are Asperger syndrome, where there are delays in cognitive development and language, and Pervasive developmental disorder. Childhood disintegrative disorder, also known as Heller's syndrome and Rett syndrome are very close to autistic spectrum disorders as to certain signs, such as impaired social interaction.

ASD has a strong genetic basis, although the genetics of autism are complex and it is unclear whether it is explained more by rare mutations with major effects, or by rare multigene interactions of common genetic variants. The arising complexity is due to interactions among multiple genes, the environment, and epigenetic factors which do not change DNA but are heritable and influence gene expression (Abrahams & Geschwind, 2008; Buxbaum, 2009). Several lines of evidence point to synaptic dysfunction as a cause of autism. All known teratogens related to the risk of autism appear to act during the first eight weeks from conception, and though this does not exclude the possibility that autism can be initiated or affected later, there is strong evidence that autism arises very early in development (Arndt *et al*., 2005).

An association between autism and prenatal **thalidomide** was found in Sweden study of autistic poeple (Strömland *et al*., 1994; Strömland *et al*., 2002). Compared to therate of autistic children in Sweden (approximately 1/1000), there was a highly significant increase in thalidomide victims, about 5%. Animal studies showed that maternal administration of thalidomide or valproic acid caused disruption of early serotonegic neuronal development and neurobehavioral alterations in amanner that is, in part, consistent with human autism (Miyazaki *et al*., 2005; Narita *et al*., 2010). Other factors that have been claimed to contribute to or exacerbate autism, include **infectious disease, heavy metals, organophosphates, solvents, diesel exhaust, phthalates** and **phenols** used in plastic products, **pesticides, brominated flame retardants, alcohol, smoking, illicit drugs, vaccines**, and **prenatal stress** (Newschaffer *et al*., 2007; Kinney *et al*., 2008). Moreover, a **hyperserotonemia** in pregnancy du to SSRI treatment might be one of the reasons of the increasing frequency of autism in the human population (Hadjikhani, 2009). Today children are surrounded by thousands of synthetic chemicals. Two hundred of them are neurotoxic in adult humans, and 1000 more in laboratory models. Yet fewer than 20% of chemicals have been tested for neurodevelopmental toxicity. Prevalence, genetic, exposure, and pathophysiological evidence all suggest a role for environmental factors in the inception and lifelong modulation of ASD. Expanded research into environmental causation of autism is however needed (Herbert, 2010; Landrigan, 2010).

**Attention-Deficit Hyperactivity Disorder** (ADHD) is a neurobehavioral developmental disorder. It is primarily characterized by “the co-existence of attention problems and hyperactivity, with each behavior occurring infrequently alone” and symptoms starting before seven years of age. ADHD is the most commonly studied and diagnosed psychiatric disorder in children, affecting about 3% to 5% of children globally and diagnosed in about 2% to 16% of school aged children. It is a chronic disorder with 30% to 50% of individuals diagnosed in childhood continuing to have symptoms into adulthood. Adolescents and adults with ADHD tend to develop coping mechanisms to compensate for some or all of their impairments. A specific cause of ADHD is not known. There are, however, a number of factors that may contribute to ADHD. They include genetics, diet and social and physical environments.

Adverse impacts of chronic or intermittent **hypoxia** on development, behavior, and academic achievement have been reported in many well-designed and controlled studies in humans (Bass *et al*., 2004). An experimental study on rats showed that perinatal asphyxia (non-sophisticated caesarian section model used) on day 20 of gestation in duration of 20 min resulted in hyperactivity in repeated open field test. However, the hyperactivity manifested gradually during the experiment. In the first session, the rats exhibited lower activity compared to controls, indicating their neophobia in anew environment. This behavioral pattern resembled hyperactive profile in ADHD children. They are behaviorally inhibited in an unknown environment and highly active in familiar surroundings (Dubovicky *et al*., 2007; 2008). Global brain injury produced by neonatal hypoxia also produced hyperactivity, as did hippocampal injury produced by ontogenetic exposure to **X-rays**, and cerebellar injury produced by the developmental treatments with the antimitotic agent **methylazoxymethanol** or with **polychlorinated biphenyls** (Sable *et al*., 2009). More recently, developmental exposure to **nicotine** has been implicated in childhood hyperactivity (Kostrzewa *et al*., 2008; Kable *et al*., 2009).

## Conclusion

Maintenance of the optimal fetal environment is the key factor of the future quality of life. Conditions like inadequate nutrition and oxygen supply, infection, hypertension, gestational diabetes or drug abuse by the mother, expose the fetus to a non-physiological environment. In conditions of severe intrauterine deprivation, there is a potential loss of structural units within the developing organ systems affecting their functionality and efficiency. Extensive human epidemiologic and animal model data indicate that during critical periods of prenatal and postnatal mammalian development, nutrition and other environmental stimuli influence developmental pathways and thereby induce permanent changes in metabolism and chronic disease susceptibility (Bezek *et al*., 2008). There is epidemiological and experimental evidence that environmental factors influence a diverse array of molecular mechanisms and consequently alter disease risk not only for metabolic syndrome and cardiovascular diseases, insulin resistance and diabetes mellitus, osteoporosis, asthma and immune system diseases but also for psychiatric and neurobehavioral disorders (Rinaudo & Lamb, 2008; Bezek *et al*., 2008).

Fetal and early childhood exposures to industrial chemicals in the environment can damage the developing brain and can lead to neurodevelopmental disorders, such as autism, ADHD and mental retardation. We can speak of "a silent pandemic". As it was mentioned in the editorial in the Nature (2010), the beginning of the 21^st^ century is becoming a decade for psychiatric disorders. Mental disorders affect more than 27% of adult Europeans every year. They are the main cause of suicides in European Union. Economic issue of mental and behavioral disorders is also inconsiderable, treatment of these disorders can cost up to 4% of GDP (Krištofiɩová, 2008). Researchers have found that about 200 industrial chemicals have the capacity to damage the human brain, and they conclude that chemical pollution may have harmed the brains of millions of children worldwide (Labie, 2007). Nevertheless, the toxic effects of industrial chemicals on children have generally been overlooked.

Further knowledge on possible unfavorable effects of chemical compounds as well as other harmful factors on the developing brain is needed. Serious epidemiological studies along with experiments by using appropriate animal models can help to elucidate possible risks for the developing organism and may decrease the frequency of mental and neurobehavioral disorders in the human population.
